# The efficacy of Kinesio taping in patients with post-stroke dysphagia: A meta-analysis

**DOI:** 10.1097/MD.0000000000037491

**Published:** 2024-03-15

**Authors:** Xiaomei Li, Hejia Cai, Ke Tang, Fangcun Li

**Affiliations:** a Guangxi University of Chinese Medicine, Nanning, China; b Guilin Municipal Hospital of Traditional Chinese Medicine, Guilin, China; c College of Physical and Health Education, Guangxi Normal University, Guilin, China.

**Keywords:** dysphagia, efficacy evaluation, Kinesio taping, meta-analysis, stroke

## Abstract

**Background:**

Dysphagia, or swallowing dysfunction, is a commonly observed complication among stroke patients, which has been associated with increased mortality rates. The treatment of post-stroke dysphagia encompasses various therapeutic approaches, and Kinesio taping has recently emerged as a potentially effective intervention. This study aims to evaluate the potential benefits of Kinesio Tape in improving dysphagia symptoms in individuals who have experienced a stroke.

**Methods:**

his study searched PubMed, Embase, The Cochrane Library, Web of Science, Wanfang Medical Database, CBM, CNKI, and Wipro VIP databases. Randomised controlled trials on the effect of intraosseous patches on the recovery of swallowing function in stroke patients were collected according to the inclusion and exclusion criteria. The search was conducted from from the date of database construction to June 2, 2023. Included trials were assessed using the Cochrane Risk of Bias tool. Meta-analyses were performed using ReviewerManager 5.4.1, and publication bias tests were performed using stata17.

**Results:**

A total of 12 randomized controlled trials consisting of 724 patients were included in the analysis. The results showed that the effective rate of Kinesio taping [RR = 1.27, 95% CI (1.16, 1.39), *P* < .00001], swallowing function score [MD = 0.78, 95% CI (0.45, 1.11), *P* < .00001], and quality of life score for patients with swallowing disorders [MD = 21.68, 95% CI (8.47, 36.90), *P* = .001] were all superior to those of the controls.

**Conclusion:**

Kinesio taping have been shown to improve swallowing function and nutritional status in patients with dysphagia in the pharyngeal phase.

## 1. Introduction

Stroke is the second foremost cause of death worldwide and the leading cause of disability.^[1]^ Dysphagia is a prevalent occurrence after stroke, affecting between 50% to 80% of stroke survivors. Dysphagia-related malnutrition and dehydration can increase the risk of respiratory infections and pneumonia, with up to a 50% associated mortality ratewing function within a few weeks of onset, 11% to 50% of stroke patients continue to experience difficulties with swallowing.^[2,3]^ Post-stroke dysphagia (PSD) not only jeopardizes the safety and efficacy of food intake but also increases the risk of pneumonia and malnutrition.^[4]^ If left untreated, dysphagia can negatively affect mental health, hinder independence, and slow recovery. Therefore, it is essential to address effective treatment options that restore swallowing function after stroke.

Various current treatments for PSD include behavioral interventions, medication, and physical stimulation. However, some clinical treatments, like internal drug therapy, may produce adverse effects. Kinesio taping serve as excellent non-pharmacological treatments with significant benefits in boosting lymphatic return and local blood circulation, and reducing fatigue and pain in soft tissues. Kinesio taping are widely applied in neurological rehabilitation. Recently, a systematic evaluation has reported good efficacy of the Kinesio taping in treating upper and lower limb dysfunction in stroke patients.^[3,4]^ While Kinesio taping have been used to treat PSD, no analysis has studied the use of inotropic patches for post-stroke swallowing disorders. Therefore, this meta-analysis aims to evaluate the effectiveness of inotropic patches for treating swallowing disorders in stroke patients in order to provide guidance for PSD treatment.

## 2. Materials and methods

### 2.1. Literature search strategy

The study adhered to international guidelines for writing meta-analyses pertaining to the selection and utilization of research methods. The protocol was registered on the international prospective register of systematic reviews (http://www.crd.york.ac.uk/PROSPERO, accessed on June 12, 2023) with the registration number CRD42023431872.

PubMed, Embase, The Cochrane Library, Web of Science, wanfang data, CBM, CNKI and weipu data were searched. The search was conducted from the date of database construction to June 2, 2023. the search was conducted using a combination of subject and free terms. Literature searches were conducted using a combination of subject terms and free terms. The search terms included “stroke,” “Dysphagia,” “Kinesio tape,” “swallowing disorder.” To obtain all randomized controlled trials with Kinesio taping for the treatment of patients with post-stroke dysphagia, we also tracked the literature retrieved to supplement the relevant literature. The complete search strategy for each database is shown in Table 1.

**Table 1:** General characteristics of the detailed study.

Reference	Country	Sample size (T/C)	Mean age, yr (T/C)	Treatment methods		Dosage	Outcome
				T	C		
Huan, C. 2020^[4]^	China	32/30	66.25 ± 11.48/66.70 ± 12.25	KT and II	II and IV	2 d/t, 24 d	①, ②
Shaohua, W. 2018^[5]^	China	30/30	64.65 ± 5.34/63.01 ± 6.27	KT and II	II	24 min/t, 1 t/d,4 wk	①, ④
Xuezhen, Z. 2019^[6]^	China	56/52	62.34 ± 8.64/13.89 ± 4.1	KT and II	II	24 min/t, 1 t/d, 8 wk	①, ③
Xiufang, Y. 2020^[4]^	China	30/30	69.35 ± 5.43/67.61 ± 5.21	KT and I and II	I and II	3 wk	⑤
Xiaosong, Z. 2021^[6]^	China	23/22	19.6 ± 3.5/19.8 ± 4.0	KT and II	II	1 t/d, 6 t/wk, 4 wk	①, ②
Peiwan, L. 2016^[5]^	China	20/20	63.10 ± 5.97/63.95 ± 7.05	KT and II	II	24 min/t, 1 t/d, 2 wk	⑤, ⑥
Qinghua, Y. 2021^[7]^	China	46/45	60.3 ± 3.1/59.6 ± 2.9	KT and II	II	12 h/t, 1 t/d, 15 d	②, ④
Huan, C. 2019^[8]^	China	36/36	67.3 ± 8.37/69.8 ± 10.96	KT and II	II	12 h/t, 1 t/d, 15 d	①, ②
Jing, G. 2023^[9]^	China	32/32	64.60 ± 5.05/66.66 ± 4.56	KT and I	I	1 t/2 d, 30 d	③, ⑥, ①
Wei, W. 2020^[10]^	China	25/25	55.45 ± 5.13/56.23 ± 4.36	KT and II	II	30 min/t, 1 t/d, 2 mo	⑤, ①
Ays ¸e Güleç 2021^[11]^	China	12/12	66.3 ± 8.4/67.0 ± 4.6	KT and II and III	II and III	24 min/t, 1 t/d, 3 mo	⑥
Weili, W. 2020^[12]^	China	25/25	55.73 ± 1.86/55.06 ± 2.39	KT and II	II	24 min/t, 1 t/d, 15 d	①

① Clinical symptom effective rate; ② Cany Island Ichiro swallowing curative efficacy evaluation.

C = control group, GUSS = gugging swallowing screen, I = acupuncture, II = conventional rehabilitation therapy, III = Nerve muscle electrical stimulation, IV = placebo patch, KT = Kinesio tape, RSST = repetitive saliva swallowing test, SWAL-QOL = swallowing quality of life, t = time, T = trial group, WST = water swallow test.

### 2.2. Eligibility criteria and outcome indicators

The inclusion criteria: A randomized controlled trial (RCT) of an intramuscular effect patch for the treatment of swallowing function in stroke patients; Subjects met diagnostic criteria for post-stroke dysphagia; Kinesio taping intervention in conjunction with conventional rehabilitation in the test group; Conventional rehabilitation (physical therapy, occupational therapy, etc.) for the control group; Key outcome indicators: efficiency, Ichiro Fujishima Dysphagia Rating Scale; and Secondary outcome indicators: Repetitive Saliva Swallowing Screening Test (RSST), Swallowing Quality of Life Scale (SWAL-QOL).

The exclusion criteria: literature not in Chinese or English; controls were only blank controls and physiotherapy alone; and literature with duplicate publications, incomplete data or unavailable data.

### 2.3. Literature screening and data extraction

Step 1-Import the retrieved literature into the literature management software Endnotex 9.3.1. Step 2-Exclude duplicate material. Step 3-Perform the first round of screening by reading the title and abstract. Step 4-Download the full text and perform a second round of screening to determine if the inclusion criteria are met.

Thereafter, literature screening, data extraction and quality evaluation were carried out independently by 2 researchers, CHJ and LXM. The results were cross-checked by the evaluators. In the literature screening, we first read the title to exclude irrelevant literature. We then further read the abstract and full text to determine if it was included. If required, the original study authors were contacted by email and telephone to obtain information that was not identified but was important to this study.

The information extract includes:

① Basic information about the included studies, i.e. authors, date of publication, title;
② Baseline characteristics of the study population
③ Measures of treatment;
④ Key elements of the risk of bias evaluation;
⑤ Ending indicators and outcome data.

### 2.4. Quality assessment

Two investigators independently used the Cochrane Collaboration tool to examine the risk of bias in the included studies. Six areas were included. generation of randomized protocols; allocation concealment; blinding; completeness of data; selective reporting; and other. The level of risk of bias was categorized as “low risk,” “high risk,” and “unclear.” Two researchers independently evaluated the risk of bias of the included studies and cross-checked the results.

### 2.5. Statistical analysis

Reviewer Manager 5.4.1 software was used for statistical analysis. Where outcomes included in this paper were count data, the ratio of odds ratios (OR) was used as the effect analysis statistic. If the outcomes included in the literature were continuous variables and came from the same assessment method, we used mean difference (MD) and 95% confidence interval (CI) for the statistics. If the results were not from the same assessment method, standard mean differences and 95% confidence intervals (CI) were performed. Heterogeneity between the results of included studies was quantified using *P* values combined with *I*^2^ values to determine the magnitude of heterogeneity. When the *P* ≥ .10, there was no heterogeneity between studies, whereas *P* < .10 indicated that heterogeneity existed between studies. If *I*^2^ < 50%, a slight heterogeneity between studies was indicated and a fixed effects model was used for analysis. If *I*^2^ ≥ 50%, heterogeneity existed in this study and Meta-analysis was performed using a random effects model. the level of Meta-analysis was set at α = 0.05. Egger’s test was used to assess publication bias and sensitivity analysis for studies that included more than 5 studies using Stata 17.0 software. Whether there was publication bias. Differences were considered statistically significant at *P* < .05.

### 2.6. Ethics and dissemination

This study used published data that were not linked to individuals, so does not require ethical approval.

## 3. Results

**Figure 1.**
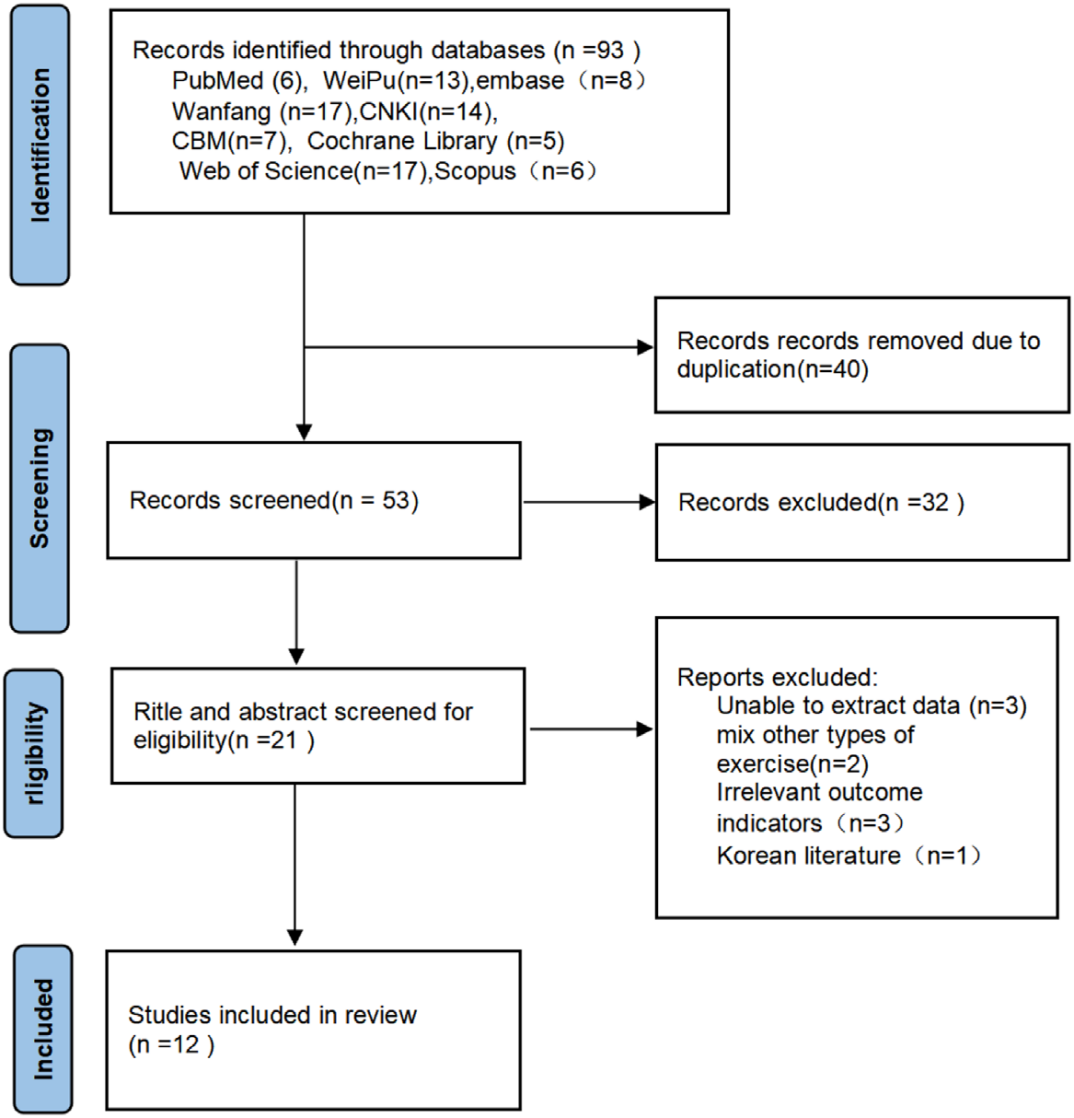
Study selection represented by the PRISMA flow chart.

A total of 93 relevant publications were obtained from the initial review, of which: PubMed (n = 14), The Cochrane Library (n = 5) Web of Science (n = 17), Scopus (n = 6), Embase (n = 8), Wan Fang (n = 17), CBM (n = 7), CNKI (n = 14), and VIP (n = 10). By excluding duplicate literature yielded 21, excluding 8 studies with main unextractable data, 2 mixed other types of campaigns, 3 with inconsistent outcome indicators and 1 Korean study, 12 RCTs including 726 patients were finally included. The literature screening process is shown in Figure 1.

### 3.1. Basic information on the included literature

**Figure 2.**
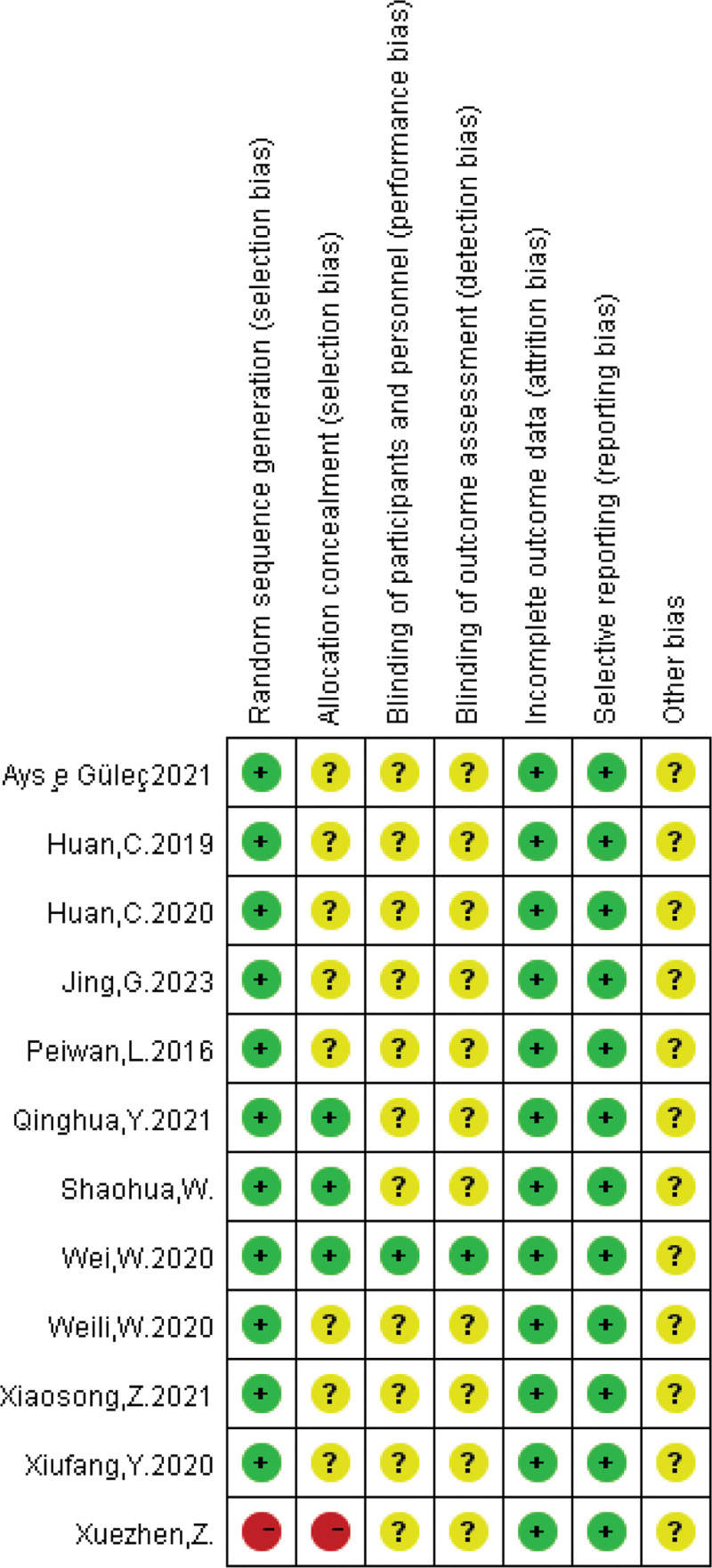
Specific risks of bias in each study.

**Figure 3.**
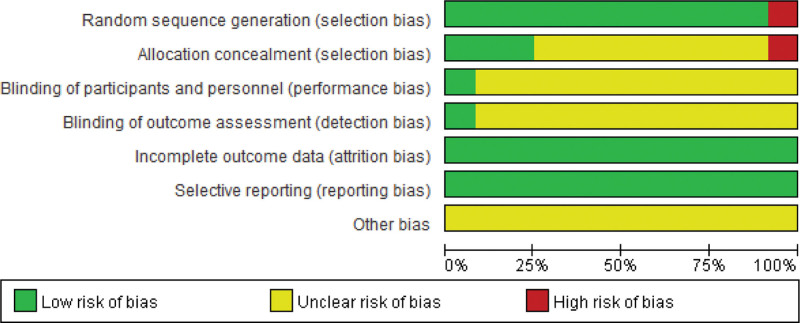
Risk of bias assessment results.

All 12 articles included in this study were randomized controlled trials (RCTs), including 3 articles that used random number tables for randomization and 6 articles that did not specify the randomization method. There was no description of allocation concealment or blinding in any of the articles. The data reported in all articles regarding the outcomes were complete. The assessment of the risk of bias in the included studies and the percentage of each bias are shown in Figures 2 and 3.

### 3.2. Meta-analysis results

Clinical efficacy was analyzed using a fixed effects model based on the results of the heterogeneity assessment. A random effects model was used to analyze swallowing function and quality of life.

**Figure 4.**
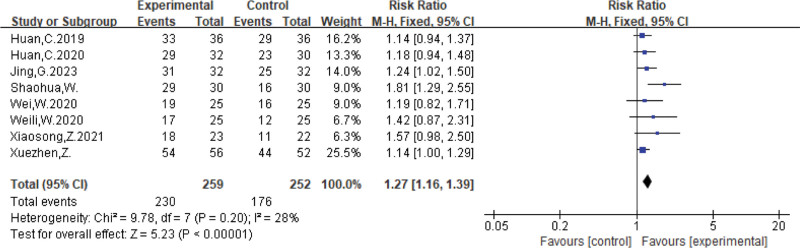
Meta-analysis forest plot of clinical effectiveness of the 2 groups of patients.

In the literature included in this study, a total of 8 articles^[4–11]^ containing 511 participants assessed the effectiveness rate. We found a low test for heterogeneity in response rates (*P* = .20, *I*^2^ = 28%), analyzed using a fixed effects model [RR = 1.27, 95% CI (1.16, 1.39), *P* < .00001]. The results showed that the clinical symptom efficiency ratio of the treatment in the Kinesio taping group was better than that of the control group. The results are shown in Figure 4.

**Figure 5.**
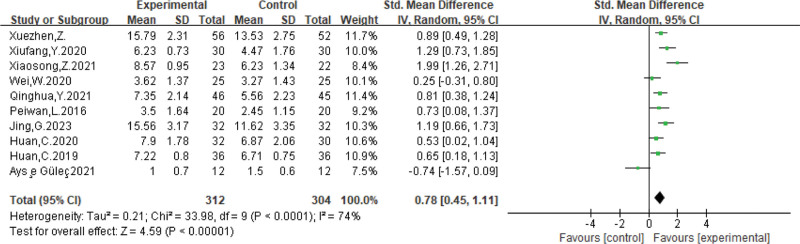
Meta-analysis of the effect of swallowing function after inotropic patch intervention.

Based on 10 articles in the literature^[4,6,8–10,12–15]^ that included 616 participants and 4 indicators to evaluate swallowing function, statistical analyses were conducted using standardized mean genus differences. We found heterogeneity in swallowing function (*P* < .0001, *I*^2^ = 74%) using a random effects model analysis, [MD = 0.78, 95% CI (0.45, 1.11), *P* < .00001] and the results were statistically significant, which suggests that the swallowing function ratio was better in the Kinesio taping group treatment than in the control group, the results are shown in Figure 5.

**Figure 6.**
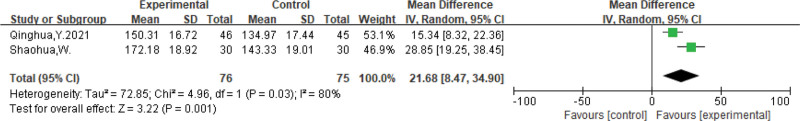
Meta-analysis of the impact on quality of life after inotropic patch intervention.

Based on 2 studies^[5,16]^ that included 151 participants. We found heterogeneity in SWAL-QOL (*P* = .03, *I*^2^ = 80%), analyzed using a random effects model [MD = 21.68, 95% CI (8.47, 36.90), *P* = .001]. The results were statistically significant, meaning that the Kinesio taping group was effective in improving the level of quality of life levels compared to the control group. The results are shown in Figure 6.

### 3.3. Publication bias and sensitivity analysis

Begg’s test was used to analyze publication bias for outcome indicators involving 5 or more randomized controlled trials. The results showed no publication bias for efficiency (*t* = 2.29, *P* = .062, *P* > .05), swallowing function scale (*t* = −0.39, *P* = .703, *P* > .05).

We performed sensitivity analyses on efficiency and swallowing function by elimination on a case-by-case basis in the included randomized controlled trials. There was no significant change in the combined results after the exclusion of any of the RCTs and the results were stable.

### 3.4. Analysis of complications

There are 2 reports^[9,16]^ the overall incidence of complications such as malnutrition, aspiration pneumonia, vomiting reflux and aspiration was lower in the KT group.

## 4. Discussion

With the continuous innovation and progress of rehabilitation methods, intramuscular patches have been included in post-stroke rehabilitation treatment plans to improve the efficacy of rehabilitation treatment.^[16,17]^ Intramuscular patch is a physical therapy method that does not contain any medicinal ingredients and has ultra-thin breathable tape with excellent elasticity. By applying this patch to the surface of the body, it can increase sensory input, relax, or promote soft tissue functional activity, improving various aspects of the patient’s motor and sensory functions. Intramuscular patches can jointly control and maintain muscle tone by activating skin receptors, enhancing peripheral incoming signals, providing feedback and regulating the central nervous system, joints, and muscles.^[18]^ They can also relax the muscle fascia in areas of overcompensation, and regulate the structural stability and muscle tone of the body through the fascia network to achieve posture control. This study indicates that swallowing function in patients with post-stroke dysphagia is significantly improved after intramuscular patch intervention, while reducing the incidence of lung aspiration in patients. Based on previous research, the mechanism by which intramuscular patches improve muscle function is to stimulate skin receptors, increase sensory input, and cause neural reflexes, thereby recruiting more motor units during maximum muscle contraction, increasing subcutaneous space, and promoting circulation, both of which can activate muscles.^[19]^ The research results of Chen et al^[8]^ indicate that intramuscular patches have no significant therapeutic effect on patients with oral swallowing disorders. The reason may be that the problem with oral swallowing disorders is not only in the facial muscles, but mainly in the inability of the tongue to stir food or transport food to the base of the tongue, achieving the goal of inducing swallowing initiation.^[18]^ However, intramuscular patches improve the contraction ability of facial muscles and cannot improve the stretching ability of the tongue. The use of intramuscular patches has a significant effect on patients with swallowing disorders during the pharyngeal period. Multiple research results have confirmed this viewpoint.^[5,12,13]^

Studies have shown^[10,20]^ that patients with swallowing disorders after stroke can affect their quality of life. The results of this study indicate that intramuscular patches can improve swallowing function and nutritional status in patients with swallowing disorders during the pharyngeal phase. This provides higher accuracy evidence for the clinical application of this treatment method. This study included 12 randomized controlled trials, and the results were consistent with each randomized controlled trial included, confirming the efficacy of intramuscular patches in treating post-stroke dysphagia. However, after careful analysis of each RCT, they all have varying degrees of defects and biased results. Therefore, they will reduce the quality of the results. The main reason for bias is speculated to be due to differences between included studies. This may be due to the different training methods adopted by different institutions, and some studies lack reports on hidden allocation mechanisms and dropout criteria, making it impossible to estimate and exclude possible impacts. In addition, there is limited research in China and other countries on the treatment of post-stroke dysphagia with intramuscular patches, and there is a lack of high-quality, large-scale multicenter studies, resulting in low quality of evidence. This test is essentially a screening method for swallowing difficulties, with high screening reliability, validity, and effectiveness. However, it is not superior to other methods in evaluating patient swallowing function and clinical efficacy. We suggest using high-precision evaluation methods, using evaluation scales with higher reliability and validity, and using gold standard methods (i.e. VFSS and FEE) to evaluate outcome indicators. Sample size estimation should also be conducted to ensure the inclusion of a sufficient number of samples to improve research quality and evidence accuracy.

Limitations of this study: The implementation frequency, duration of treatment, starting and ending points of patch fixation, and anchor (starting point) width of intramuscular patches are not completely the same, which may lead to certain clinical heterogeneity; Different forms of rehabilitation training and therapists may affect research results; Some studies included have not been clearly reported or randomized trials, methods of random allocation, allocation concealment, and blinding, resulting in selection, implementation, and measurement biases; The reports of adverse reactions are limited, although due to the small number of studies included, this finding may not indicate the true clinical situation; and The proportion of foreign literature included in the study is relatively small, which limits the universality of our research results. If some publications from around the world can be included, it will make the review an international or global review. Unfortunately, we were unable to find any relevant literature in the region, which makes this review unable to generate global or international impact.

## 5. Conclusions

In summary, the results of this study demonstrate the effectiveness of the inotropic patch in treating patients with pharyngeal dysphagia and improving swallowing function and nutritional status in patients with pharyngeal dysphagia. Due to the limitation of the number and quality of included studies, the above findings have yet to be validated by more high-quality studies.

## Author contributions

**Conceptualization:** Xiaomei Li.

**Data curation:** Hejia Cai.

**Formal analysis:** Hejia Cai.

**Funding acquisition:** Xiaomei Li.

**Methodology:** Xiaomei Li.

**Project administration:** Fangcun Li.

**Resources:** Ke Tang.

**Software:** Xiaomei Li , Hejia Cai.

**Supervision:** Fangcun Li.

**Visualization:** Hejia Cai.

**Writing – original draft:** Xiaomei Li, Hejia Cai.

**Writing – review & editing:** Fangcun Li.
